# Corona and value change. The role of social media and emotional contagion

**DOI:** 10.1007/s10676-020-09545-z

**Published:** 2020-07-21

**Authors:** Steffen Steinert

**Affiliations:** Department of Values, Technology and Innovation, Faculty of Technology, Policy and Management, Delft University of Technology, Delft, The Netherlands

**Keywords:** Social media, Emotions, Emotional contagion, Values, Value change

## Abstract

People share their emotions on social media and evidence suggests that in times of crisis people are especially motivated to post emotional content. The current Coronavirus pandemic is such a crisis. The online sharing of emotional content during the Coronavirus crisis may contribute to societal value change. Emotion sharing via social media could lead to emotional contagion which in turn could facilitate an emotional climate in a society. In turn, the emotional climate of a society can influence society’s value structure. The emotions that spread in the current Coronavirus crisis are predominantly negative, which could result in a negative emotional climate. Based on the dynamic relations of values to each other and the way that emotions relate to values, a negative emotional climate can contribute to societal value change towards values related to security preservation and threat avoidance. As a consequence, a negative emotional climate and the shift in values could lead to a change in political attitudes that has implications for rights, freedom, privacy and moral progress. Considering the impact of social media in terms of emotional contagion and a longer-lasting value change is an important perspective in thinking about the ethical long-term impact of social media technology.

## Introduction

The current Coronavirus pandemic is an emotionally taxing time and people have a tendency to express and share their emotions, especially on social media platforms. Evidence seems to suggest that it is primarily negative emotions, like fear and anxiety, that are shared in times of crisis. The idea pursued in this paper is that emotions and their spread on social media play a big role for a potential value shift in the wake of the recent Coronavirus pandemic. (The Coronavirus, or SARS-CoV-2, is the virus that causes the disease COVID-19). The expression of negative emotions and feelings via social media, thereby reaching a lot of other people, could lead to an emotional contagion creating a negative emotional climate. This development is exacerbated by the fact that social media rewards emotionally charged messages.

Studies in psychology and sociology show that people adapt their values to circumstances. Furthermore, values are internally structured so that when certain values increase in importance, the values that express opposite goals decrease in importance. When people are in a threatening situation or perceive a situation to be threatening, their values shift towards values emphasizing the security and conformity. A pervasive negative emotional climate facilitates the perception of threat and could thus contribute to a change in personal value towards values that emphasize security and stability of society. Because personal and political values are related, threat-based change in personal value will likely foster a change in political values. In turn, the changed political values will lead to preferences of policies that focus on security, stability, and conformity. This political change could come to the potential detriment of rights and civil liberties because in times of perceived threat, people are more willing to give up said civil liberties. The account presented here of how emotional climate relates to value change also has normative implications for how we approach decision-making about the introduction of technologies that are supposed to remedy some of the consequences of the crisis. Again, because people want social stability, health, and economic welfare to be secured, they could be willing to accept technologies that promise that. Some of these technologies carry ethical risks, and we should make sure that ethically risky technology is not hastily introduced out of an emotional climate.

Although focused on the Coronavirus pandemic, the paper makes a larger contribution. Understanding the link between emotions, value change, and information technology can help to better grasp the role of technology in potentially socially disruptive long-term changes.

## Crisis, negative emotions and social media

Emotions are based on values and concerns, that is, the things people care about (Roeser and Todd 2014; Todd 2014). According to the appraisal theory of emotion, emotions are responses that reflect a person’s assessment of how significant something in the environment is for their well-being (Moors et al. 2013). Similarly, the philosopher Robert Roberts has argued that emotions are concern-based construals (Roberts 2003). Concerns are the things in life that people care about, including their needs and their attachments to things or other people. Amongst other aspects, people have a concern for their well-being and their bodily integrity. Furthermore, people are also concerned about and attached to other people. Based on these attachments and interests (aka concerns), emotions signal that something in the situation affects something a person is concerned about. For instance, because a parent cares for their child, fear is usually the response when the well-being of the child is threatened.

People usually care deeply about their health and the health of the people close to them. People also care about job security and personal freedom. Many people perceive the current Coronavirus pandemic as a threat to all of these things. With social distancing and quarantine as legal requirements in many countries, freedom is limited, likely leading people who are less afraid of health consequences to experience other negative emotions like anger or frustration.

Another important feature of emotions, besides appraisal, is that they have particular action-tendencies (Frijda 1986). When we care about something, we are motivated to pursue courses of action that support or avoid harm to what we care about. Thus, current fear for their lives, health, and livelihood will likely motivate some people to take protective actions. What is important here is that people perceive there to be a threat, whether or not the threat exists. A perceived threat is enough to motivate people. As will become clear later, the spread of negative emotions via social media can foster the perception that the threat is greater than it is.

People tend to share emotions with others and are especially prone to sharing intense emotions (Rimé 2009). In crises or traumatic situations, like natural disasters, accidents, or terrorist attacks, people experience a range of intense emotions. In trying times, social media is a popular medium for many people to share their thoughts and emotions. For example, immediately after the 2004 terrorist attack in Spain, there was a steep increase in communication about the event, including the sharing of emotions (Rimé et al. 2010).

Because people share their feelings on social media, scientists use it to gauge the emotions of people related to situations of crisis. They found that negative emotions, like fear, anger, sadness, and a feeling of insecurity, prevail in these situations. For instance, in the two weeks after the terrorist attacks in New York City in 2001, people expressed more negative emotions in online diaries (Cohn et al. 2004). Anger was a leading emotion in the public’s expression on Twitter regarding the disappearance of flight MH307 (Yeo et al. 2020). In an analysis of Tweets during hurricane Sandy in 2012, a huge number of Tweets belonged to the anger and fear category (Brynielsson et al. 2013). Finally, in a study including over 60.000 Twitter users after the terrorist attack in 2015 in Paris, Garcia and Rimé (2019) found collective expressions of sadness and anger (but also long-term increase in expressions of solidarity). Expressing emotions online does not mean that these emotions are inauthentic or do not represent what people really feel. Although some people could misrepresent their emotions online, there is no evidence that misrepresentation is pervasive.

In the current Coronavirus pandemic, people experience that a lot of the things that they value are threatened. Subsequently, people experience a lot of emotions, especially negative emotions. In a not yet peer-reviewed preprint including a dataset about the worries and emotions of UK residents collected in early April 2020, where participants had to write a short text about how they feel about the pandemic, researchers found that the prevalent emotions were anxiety, fear, and sadness (Kleinberg et al. 2020). Perhaps it is not surprising then, that on social media people express predominantly negative emotions, like fear and anxiety. Here is some of the early available evidence for the emotions that people encounter and express on social media during the Corona outbreak. (Please note that some studies are pre-prints of yet to be published papers). Looking at the link between social media exposure and mental health problems during the Corona outbreak in China, researchers found that high social media exposure is associated with a high prevalence of depression and anxiety (Gao et al. 2020). Examining data from the social media platform Weibo, other researchers found an increase in negative emotions, like anxiety, after the announcement of the disease COVID-19 (Li et al., 2020). The data also seems to indicate that people worry about their jobs and the economic situation in general. Analyzing all Corona-related Twitter activity from mid- to end-January 2020, that is the early stages of the outbreak, researchers found that almost half of the tweets expressed fear (Medford et al. 2020). Finally, one study conducted in March 2020 found that instances of fear, sadness, and disgust were prevalent worldwide with the US, the Netherlands, France, and Switzerland showing especially high levels of expressed distrust and anger (Dubey 2020).

In the following, I make the case that the sharing of negative emotions on social media could contribute to the development of a negative emotional climate in a society. This emotional climate could contribute to a change in personal values and this value change can have political ramifications. Specifically, there is a link between personal values and political preferences. When people perceive their values to be threatened, they prefer policies that protect these values and are more inclined to accept measures that limit their civic freedom. People may also be quicker to accept proposed technological remedies to the crisis, without proper deliberation of the ethical risks. To understand how such a value change facilitated by online emotion expression and contagion can occur, I will first introduce emotion sharing and emotional contagion.

## Social media and emotional contagion

In the last section I presented evidence that in traumatic situations and crises, people use social media to share their emotions. On social media emotions, much like in the offline world, emotions can spread from one person to another. This spread is known as emotional contagion. People are affected by the emotions of others and emotions can spread from one person to another. Emotion contagion refers to the phenomenon that people’s emotions become similar to other people’s emotions because they were exposed to the emotions of these other people. Some authors have likened emotions to infectious diseases that spread in social networks over an extended period (Hill et al. 2010). People are usually not aware of emotional contagion. Furthermore, emotional contagion has consequences that extend beyond how people feel because emotions influence how people think and act (Barsade et al. 2018). The sharing of emotions can lead to emotion sharing feedback loops (Garcia and Rimé 2019). That is, people talk or write about an event in reaction to how other people talk or write about the event.

The emotional feedback loop and emotional contagion are accelerated by digital technology and social media (Hill et al. 2010). Social media makes it easy to express and communicate emotions to people beyond the immediate social circle. This also increases the receivers of emotional contagion. Furthermore, in times of Coronavirus pandemic and social distancing requirements, many people spend more time in front of a screen, likely increasing emotion sharing and emotional contagion online.

In the remainder of the text, I will use the terms digital emotions and online emotions to refer to emotions that are expressed online or experienced about the content posted on the internet, especially social media. That means that digital emotion or online emotion is not a new kind of emotion. Importantly, in contrast to emotions that spread via face-to-face communication, digital emotions are technologically mediated emotions. That means the way that technology contributes to the way that emotions are shared and distributed. Compared to offline emotion contagion, digital emotion contagion describes the phenomenon that the receivers’ emotions become more like the emotions of the people that posted emotional messages (Goldenberg and Gross 2020). Again, emotional contagion explains how the transmitted emotions of individuals can grow into digital group emotions.

Social media platforms contribute to the spread of emotions online and subsequent emotional contagion. This has also to do with the business model of digital media companies and how they design their social media platforms. For instance, it makes sense for digital companies to try to promote emotion expression because emotions keep people engaged on the platforms and engagement means more opportunities to present ads and gather data. The way that emotion captures attention is an important part of the explanation of why emotional content goes viral on social media (Brady et al. 2020). Indeed, on social media emotional information spreads more quickly than information that is not related to emotions. For instance, Twitter messages about cancer that included joy, sadness, and hope are liked more than others, and tweets that contain joy and anger are retweeted more than others (Wang and Wei 2020). Furthermore, the presence of emotional-moral words in social media messages increase their spreading substantially (Brady et al. 2017), and digital media platforms seem to exacerbate content that induces outrage (Crockett 2017). The way that social media platforms operate may even intensify the negative aspects of outrage, like harassment or potentially anger, instead of turning outrage into a force of social progress (Brady and Crockett 2019). The interest of digital media companies in people’s emotions is also highlighted by the now infamous emotion manipulation study by Facebook in 2014 (Kramer et al. 2014), where users' emotions where manipulated through the emotional content of their news-feed to gauge emotion contagion through the social network. Although there is some evidence that on social media platforms people usually share positive emotions more often than negative emotions. However, anger seems to be the exception (Goldenberg and Gross 2020) and some studies indicate that social media posts expressing anger are more likely to spread among users than posts expressing joy or sadness (Fan et al. 2014).

People’s prevalent expression of negative emotions, like anger, fear, sadness, or disgust, during the current Corona crisis may lead to an accelerated spread of negative emotions via emotional contagion. Consequently, the emotional contagion mediated by social media may contribute to a long-lasting change in how people emotionally relate to the world.

The idea of an *emotional climate* is helpful here to think about the change in how individuals emotionally relate to the world and other people. Joseph de Rivera (1992) proposes that an emotional climate can be distinguished from emotional culture and emotional atmosphere. An emotional culture comprises the cultural codes and symbolic inventory by which emotions are expressed and regulated. For instance, emotion rules, emotion norms, and emotion narratives are part of the emotional culture. Emotional atmospheres can occur when people jointly relate emotionally to a short-term event. For instance, the event of a political speech can have a particular emotional atmosphere. In contrast to an emotional atmosphere, an *emotional climate* is a longer-lasting way that people of a social community or society emotionally relate to the world and one another. In contrast to an emotional atmosphere, which is more transient and event-related, an emotional climate depends on pervasive socio-political and socio-economic conditions. Examples for these conditions that affect many people are social inequality, repressive leadership, poverty but also natural disasters and widespread disease. There are positive and negative emotional climates. A positive emotional climate may occur in the aftermath of political revolution and in times of economic growth. For example, de Rivera (1992) describes a shift from the emotional climate of fear to the emotional climate of hope in Chile at the end of the military dictatorship.

In the following sections, I show how a change towards a negative emotional climate may contribute to a change in personal values, which in turn has political ramifications.

## Value change and crisis

To understand how emotions may contribute to a change in people’s values, it is important to understand how values relate to each other and how value change can occur.

Values are conceptions of “the desirable” that guide social actors, like people and groups, in their selection of actions and that influence the evaluation of actions and state of affairs (Kluckhohn 1951, p. 395; Rokeach 1973; Schwartz 1999, p. 24). We know from psychology and sociology that values are not static but can change. Value change can occur on the individual as well as the collective or social level. For instance, lasting individual value changes have been observed concerning major life transitions like migration to a new country (Bardi et al. 2014). Education may also contribute to long-lasting value change. For instance, completing an MBA Program can lead to an increase in self-oriented values and a decrease in other-oriented values (Krishnan 2008).

To understand value change, it is helpful to consider how values are internally related. According to the influential Schwartz theory of basic values (Schwartz et al. 2012), there are ten broad personal values. These values can be distinguished by their principal goal or motivation. For instance, the value of security has as its defining goal the safety and stability of society, relationships, and the self. The defining goal of the value of hedonism is pleasure and sensuous gratification for oneself. The internal structure of all values, that is how they relate to each other, is determined by how compatible the values are to each other. Being motivated to pursue one value can conflict with another value. You cannot pursue conflicting values in one single act. For instance, the value of hedonism and the values of conformity or tradition do not share broad motivational goals. The first emphasizes pleasure for oneself and the second emphasizes self-restraint so as not to violate social norms and expectations. Other values, however, emphasize similar goals and are motivationally compatible. For instance, the values of conformity and security are compatible because they both share similar goals that require the subordination of the self to social expectations.

So, values are related to one another based on their motivational compatibility and incompatibility. Due to this structure of how values relate to one another, when one value increases in importance, the opposite values decrease in importance. For instance, when people perceive a threat, the importance of self-protection values, like security and tradition, increases. What decreases is the importance of oppositional values that can be called self-transcendence values (Schwartz et al. 2012). Self-transcendence values, like universalism and benevolence, emphasize the concern for the welfare and interest of other people. That oppositional values decrease/ increase in their importance could be observed in Finish students after the terrorist attacks of September 11, 2001 (Verkasalo et al. 2006).

The change of values is likely based on adaptive processes that adjust values to social, economic, and political circumstances (Schwartz and Bardi 1997). For example, looking at the value orientation of Turkish youth from 1989 to 1995, Çileli (2000) found that people adapt their value orientation to the socio-economic changes in the Turkish society: With economic improvements, young people changed to a more individualistic and competitive value orientation. Values seem particularly sensitive to the threat to individual well-being, such as economic insecurity, and some authors have argued that people’s values are shaped by how secure their survival is (Inglehart 2018). When existential survival is secure, as in times of a growing economy and job security, values prevail that are related to openness to change, diversity, and novel ideas. In contrast, in times of increased economic inequality and threatened livelihood, people concentrate on themselves, and values related to economic security become more important. For example, in a cross-national and comparative study of the change in value priorities of young Europeans after the global financial crisis, Sortheix et al. (2019) found a shift from values emphasizing growth and self-expansion, like hedonism, towards values related to self-protection, like security and tradition.

## Emotional climate in times of Corona

In the current Corona crisis, the health, well-being, and livelihood of a lot of people are in jeopardy and we have yet to witness all the negative economic consequences of the pandemic. Putting together the adaptive feature of values, how they dynamically relate to one another, and the connection between value change and existential threats, one could expect that a certain value change is likely to occur concerning the current crisis. That is because a lot of people *perceive* the Coronavirus pandemic as a threat (which is not to deny that it is a real threat). Emotions and their spread on social media likely play a big role in such a value shift.

Emotions are a factor in the stability of values because affective support sustains collectively shared values (Maio and Olson 1998). However, unbeknownst to many people that share their emotions during the Coronavirus pandemic online, an emotional climate may develop that facilitates a change in value.

The emergence of online emotion norms is one factor that could contribute to emotional contagion and subsequent development of an emotional climate. That is because emotion norms can facilitate emotional contagion. Enabled by a recurrent confrontation with certain emotions, particularly negative emotions like fear, on social media during the Corona crisis, an emotion norm to express such emotions may arise. Emotion norms are a subset of social norms. Social norms are expectations about how people, including oneself, act in certain situations (empirical expectation), and expectations of how people should act (normative expectation) (Bicchieri 2005). Like other social norms, emotion norms involve expectations about what emotions people, and oneself, should experience and expectations about what they experience. Emotion norms govern which emotions people are supposed to experience or express in a particular situation. These norms have also been called feeling rules (Hochschild 1979) or display rules (Ekman and Friesen 1975).

Like offline social life, social media is governed by emotion norms. For instance, in the case of digital mourning, norms guide the content and form of emotional display and what type of reactions are appropriate (Wagner 2018).  Researchers could show that there are different emotion norms for different social media platforms regarding what emotions are appropriate to express (Waterloo et al. 2018).

Being confronted with ubiquitous displays of particular emotions online could create the expectation that others experience a particular emotion and that you should also experience this emotion in a particular context and express it online. Emotion norms and emotional contagion mutually enforce each other: Emotion norms may emerge from and further facilitate emotional contagion. In turn, emotional contagion strengthens emotion norms.

The interplay of emotional contagion and emotion norms, facilitated by the way social media platforms encourage emotional content, may lead to a long-lasting change in the emotional climate of a society. Recall that an emotional climate is the mutable but usually long-lasting way that people of a social community or society emotionally relate to the world and one another. Also recall that emotional climates are related to political and economic factors but emotional climates can also emerge in the wake of massive collective events, like natural disasters or a pandemic. Evidence from previous catastrophes and preliminary empirical findings for the Coronavirus pandemic suggest that predominantly negative emotions, like fear and anger, are expressed online. Fostered by digital emotional contagion and emerging emotion norms, this could lead to a negative emotional climate. This emotional climate could extend beyond particular communities and nations. Social media may even “magnify the intensity of global emotional synchrony” (Coviello et al. 2014) because it allows communication and sharing of information and emotions without direct contact and across national borders.

## Emotional climate, value change and politics

As a consequence of the Corona crisis, a change towards a more negative emotional climate, which is a climate where emotions like fear and anxiety are prevalent, may contribute to a shift in values. Part of the explanation is the link between attention and emotion. You may recall that emotions are based on concerns. Emotions put the focus of our attention on a particular thing or an aspect of a situation that is relevant to our concerns. As Michael Brady puts it, “emotions capture and consume attention” (Brady 2013, Chapter 3). Fear, for instance, directs our attention to potential danger. However, people are not forced to accept the evaluative construal of an emotion. For example, I do not have to accept that the animal is dangerous only because I experience fear. Nevertheless, an emotion bestows on us an inclination to assent to the view of the situation that the emotion presents. The fact that emotions are essentially linked to attention also explains why emotional content is more engaging on social media. Emotional content just captures our attention.

The so-called broaden-and-build theory of emotions can help explain how positive and negative emotions contribute differently to value change. According to the broaden-and-build theory of emotions (Fredrickson 2004; Fredrickson and Branigan 2005), positive emotions, like joy and happiness, broaden the scope of attention and expand the thought-action repertoire. For example, joy triggers the urge to play, and curiosity triggers exploration. Due to positive emotions, the cognition of people widens and people tend to notice a wider range of possible actions and creative ideas. In contrast, negative emotions, like fear, narrow the mindset and the thought-action repertoire, and put the focus of attention on the perceived threat and means of avoidance and survival.

Undoubtedly, the Coronavirus pandemic is a threat to a lot of things people value. Negative emotions capture attention and focus it both on the perceived threat and on the means to avoid it. The negative emotional climate, facilitated by the spread of emotionally charged messages on social media, could lead to the experience that certain values are under constant threat. This, in turn, could lead to a longer-lasting change in personal values. The abovementioned internal dynamic relation of values explains how this may come about.

As noted, values are dynamically related. The rise of importance of one value means a decline in importance of another opposite value. For example, according to this account, which has been validated multiple times, the values of security and freedom are motivationally opposed; when the importance of one increases, the importance of the other decreases. Similarly, the rise of importance of security (e.g. safety and social stability) is accompanied by a decline of the importance of values related to openness to change and tolerance. Recall that motivationally compatible values can reinforce each other’s importance. For instance, values emphasizing security and tradition are compatible with each other because they have the same underlying motivational goals.

Now, a negative emotional climate, which focuses attention on a potential threat, could increase the importance of values emphasizing security, safety, and other motivationally compatible values. Thereby downgrading the importance of oppositional values. Research seems to corroborate the idea that emotions influence a change in values in such a systematic way. The fear of a (perceived) threat of terrorism has led to a change in the cosmopolitan values of tourists towards values of security (Veréb et al. 2018). Also, perception of threat, linked to the political and economic conditions in a country, has been shown to lead to diminished tolerance (Gibson 2002).

Aristotle argued that political attitudes can be influenced by evoking emotions and the influence of emotions on political attitudes is well-documented by scientists. Negative emotions seem to be particularly powerful. For instance, mediated by negative emotions like anger and anxiety, an external threat like war can improve people’s evaluation of presidential performance. The anger and anxiety after the 9/11 attacks shifted public attitude regarding the conservative president Bush in a positive direction (Lambert et al. 2010). Anger is positively related to support for aggressive policies towards out-groups (Halperin et al. 2013). The effect of emotions seems so powerful that even if the cause of anxiety has nothing to do with politics, it can carry over to the political domain and have an influence on political beliefs (Renshon et al. 2014).

Applying the account that links emotional climate to value change and political preferences to the current Corona crisis: People’s expression of negative emotions like fear or anger on social media may lead to a more negative emotional climate facilitated by emotional contagion processes. Such a negative emotional climate characterized by people’s fear for their health and the health of others may increase the importance of values like security. This, in turn, decreases the importance of values like tolerance or caring for people outside of their immediate circles. Besides, the livelihood and economic standard of many people are in jeopardy. Fear for their livelihood motivates people to protect it, which conceivably increases the importance of values related to this protection, like the value of security or conformity.

This potential value change has political ramifications because personal values are related to political preferences. Recall the value dimension of self-transcendence. Self-transcendence values include the value of universalism, which emphasizes understanding, tolerance, and the protection of the welfare of other people. Benevolence is another self-transcendence value. Basic personal values structure and anchor political values like equality, patriotism, and civil liberties (Schwartz et al. 2010). Researchers consistently show that people who strongly value universalism favor policies aimed at equality, social justice, and social welfare, whereas people who strongly value security favor political measures aimed at safety, stability, and social order (Caprara et al. 2006).

The political value of law and order is motivationally grounded in fear of uncertainty and the (perceived) threat of a disruption of social order. An anxiety-induced broad shift in personal values and political values may lead to a broad acceptance of policy decisions that limit civil liberties and reduce social justice for the sake of stability and avoidance of threat. There is some indication that a threat to personal security prompts people to give up their rights and freedoms for greater security (Davis and Silver 2004).

A negative emotional climate and the accompanying shift in values also have normative implications for how we should go about decision-making regarding the introduction of technologies that purportedly remedy some of the adversarial effects of the crisis. To secure the values of stability and security, people may be more inclined to accept surveillance of their health by digital tracking and tracing apps despite the risks for data security and loss of privacy. In the urge to fight the pandemic, leaders should not neglect the effects of a negative emotional climate on decision making and hurriedly introduce ethically risky technologies. Some leaders, particularly business leaders, may even take advantage of such a climate to push questionable technology for economic gains. Besides using an ethical framework for the development of digital interventions to fight the Coronavirus pandemic (Morley et al. 2020), people should be put in a position to effectively evaluate the ethical benefits and drawbacks of technologies heralded as counter-measures for the pandemic. A negative emotional climate can influence this evaluation, which is why even more care is needed here.

Although a fast-moving pandemic requires quick decisions, there should be a public debate and public deliberation about the technological measures that are going to be implemented is inevitable. To make political decisions more democratic and procedurally fair, the public should be involved in the process of decision making of risky technologies. Emotions should be integrated into political decision making about potentially risky technology (Roeser and Pesch 2016). Extending this idea, public debate and responsible innovation should acknowledge both the importance and potential negative impact of emotions (Steinert and Roeser 2020) and reflect on the potential impact of emotional climates on policy decisions.

Another thing to consider is that a broad societal change in values from values emphasizing tolerance and openness towards values emphasizes security and stability could strengthen people’s preference for political measures that roll back advancements in moral progress. These political measures and accompanying social changes could be hard to reverse after the crisis is over.

One aspect of moral progress is the move towards a more inclusivist morality (Buchanan and Powell 2018). Moral inclusiveness means expanding the range of entities that are candidates for moral consideration. In contrast, an exclusivist morality only considers the in-group worthy of moral consideration. There have been various moves in history towards expanding the moral circle in this sense. For instance, full moral consideration of women and minorities, and the moral acknowledgement of at least some non-human animals. Most normative theories take moral progress seriously and most normative theories would consider the expansion of our moral concerns as an improvement of morality.

Advancement of inclusivist tendencies seems to be bound to particular socio-economic conditions, like high economic productivity and high physical security. That is why an inclusivist morality could be called a “luxury good” (Buchanan and Powell 2018, p. 210). Because inclusive morality depends on favorable conditions, the possibility of regressing back to exclusivist moral responses looms, and under less favorable conditions exclusivist tendencies will likely (re)occur. For example, fear of economic security can intensify negative outgroup attitudes and lead to aggressive responses towards out-groups to preserve economic security (Riek et al. 2006).

The epistemic context plays a crucial role here. Conditions do not have to be dire, what is important is how people *perceive* the conditions. Leaders for instance can exploit that by either misrepresenting the economic situation, by making people believe that there is a threat from an outgroup or that social cohesion is in jeopardy. Social media can affect the epistemic context. Because negative emotions influence how people perceive the Corona pandemic and its consequences, a negative emotional climate could lead to a pervasive negative perception of the situation that makes a regress to exclusivist moral tendencies possible. If we care about morality, we should care about moral progress and an inclusivist morality. That also means that we should care about the possibility of a moral regress facilitated by the emotional climate and the role that technology plays for it.

## Conclusion

To briefly recap. Emotions matter, especially in times of crisis. Here, I have made the case for how, during the ongoing Coronavirus pandemic, the sharing of emotional content on social media platforms can contribute to value change. Emotion sharing could lead to digital emotional contagion which could facilitate an emotional climate. We have reason to believe this emotional climate influences the value structure. The emotions that spread in this crisis are predominantly negative (although positive emotions do occur and should not be neglected), which could result in an emotional climate that will have a negative character. Based on the dynamic relations of values to each other and the way that emotions relate to values, the negative emotional climate could result in a societal value change towards values emphasizing security and tradition and this could have particular implications for political attitudes.

The Corona crisis puts a spotlight on social, political, and economic issues that were already present before the outbreak, like health and income inequality. Similarly, looking at the link between the Corona pandemic, emotions, and social media puts into sharp relief, once again, how social media is designed to engage us and how it rewards attention-grabbing emotional content. Technology companies are already the big winners of the pandemic because social distancing drives people online. The combination of emotional contagion and social media could lead to a change in values. Ironically, the companies that provide the platforms that contribute to emotional contagion and a potential change in value are also the ones that will benefit the most from a potential value change. In the face of a lethal pandemic, privacy may decrease in priority whereas the longing for health and security increases. As a consequence, tracking and monitoring technology in the name of health may look more attractive to people, despite potential ethical risks for privacy. If negative emotions, like fear, prevail, and people are more eager to give up privacy in the name of health and security, technology companies will reap even more benefits. So maybe on top of social distancing what is needed during a pandemic of a highly contagious disease is a little bit of ‘social media distancing’ (Carmichael 2020). Thinking about the impact of social media in terms of emotional contagion and a longer-lasting value change is an important perspective in considering both the hard to notice long-term ethical impacts that social media can have and social media’s potential contribution moral regress.
